# Current News

**Published:** 1903-03

**Authors:** 


### Current News.

### INSTITUTE OF DENTAL PEDAGOGICS.

At the recent meeting of the Institute of Dental Pedagogics, held in Chicago, December 29, 30, and 31, 1902, the following officers were elected for the ensuing year: President, J. D. Patterson, Kansas City, Mo.; Vice-President, H. R. Tileston, Louisville, Ky.; Secretary-Treasurer, W. Earl Willmott, Toronto.

*Executive Committee.*—W. H. Whitslar, Cleveland, Ohio; D. R. Stubblefield, Nashville, Tenn.; R. H. Nones, Philadelphia, Pa.

The next meeting will be held at Buffalo, December, 29, 30, and 31, 1903.

Those having new teaching appliances which they wish to bring before the Institute will communicate with Dr. William C. Foster, Baltimore, or Dr. L. S. Tenney, Chicago.

---

### THE NEW YORK INSTITUTE OF STOMATOLOGY.

The annual meeting of The New York Institute of Stomatology was held December 2, 1902. The following officers were elected for 1903: President, Dr. J. Morgan Howe; Vice-President, Dr. A. H. Brockway; Recording Secretary, Dr. F. Milton Smith; Corresponding Secretary, Dr. George A. Wilson; Treasurer, Dr. J. Adams Bishop; Editor, Dr. Fred. L. Bogue; Curator, Dr. J. G. Palmer.

*Executive Committee.*—Dr. C. F. Allan, Chairman; Dr. C. O. Kimball, Dr. S. E. Davenport.

